# Recognizing Cannabis Hyperemesis Syndrome in Pediatric Patients: Insights From a Case Report

**DOI:** 10.7759/cureus.79904

**Published:** 2025-03-01

**Authors:** Pawel Rucinski, Katarzyna Akutko, Tomasz Pytrus

**Affiliations:** 1 2nd Department and Clinic of Paediatrics, Gastroenterology and Nutrition, Medical University of Wroclaw, Wroclaw, POL

**Keywords:** adolescents, cannabis, cannabis hyperemesis syndrome, cyclical vomiting, paediatrics

## Abstract

Cannabis hyperemesis syndrome (CHS) is a condition characterized by recurrent episodes of severe nausea and vomiting associated with chronic cannabis use. This case report describes a 16.5-year-old male patient who presented with a 2-year history of recurrent vomiting episodes lasting up to 10 days, accompanied by abdominal pain and weight loss. After extensive diagnostic workup and exclusion of other causes, CHS was diagnosed based on the temporal relationship between cannabis use and symptom onset. The patient's symptoms resolved with supportive care and cessation of cannabis use. This case highlights the importance of considering CHS in the differential diagnosis of recurrent vomiting in adolescents, especially given the increasing cannabis use and potency among youth. Key aspects of CHS pathophysiology, diagnosis, and management are discussed, emphasizing the need for increased awareness among healthcare providers and a multidisciplinary approach to effectively treat this complex condition in pediatric populations. The growing prevalence of CHS in adolescents suggests the need for specific pediatric diagnostic criteria in future iterations of clinical guidelines.

## Introduction

Cannabis hyperemesis syndrome (CHS) is a rare and complex condition characterized by recurrent episodes of severe nausea and vomiting that are primarily associated with chronic cannabis use. CHS often presents diagnostic challenges due to its similarity to other gastrointestinal disorders and the paradoxical nature of its symptoms, given that cannabis is commonly used to alleviate nausea. This condition can significantly affect a patient's quality of life, leading to frequent hospitalizations and disruptions in daily activities. Understanding the relationship between cannabis use and the development of CHS is crucial for the effective diagnosis, management, and prevention of recurrent episodes. This case report provides insights into the complexities of diagnosing and treating CHS, particularly in the context of increasing cannabis use worldwide, especially in the pediatric population, and highlights the importance of considering substance use patterns in the evaluation of recurrent vomiting episodes.

## Case presentation

A 16.5-year-old patient was admitted to the Department of Pediatrics, Gastroenterology, and Nutrition, Medical University of Wroclaw, Wroclaw, for the diagnosis of recurrent vomiting. The patient reported recurring episodes of abdominal pain and unrestrained, persistent vomiting lasting up to 10 days. These episodes, which followed a similar pattern, occurred several times a year over the past two years, leading to a weight loss of about 5 kg. Between episodes, a return to complete recovery was observed with no gastrointestinal complaints.

Due to his symptoms, he was hospitalized several times in pediatric wards, and symptomatic treatment was then applied, without establishing a diagnosis. Additionally, examinations performed before admission to our clinic and gastroscopy performed four months before hospitalization, have shown features of gastroesophageal reflux disease. In an abdominal ultrasound performed on an outpatient basis two months before hospitalization, thickening of the stomach wall to 0.7 cm, with a distended duodenal bulb, was observed. Except for these findings, there were no significant deviations.

**Figure 1:**
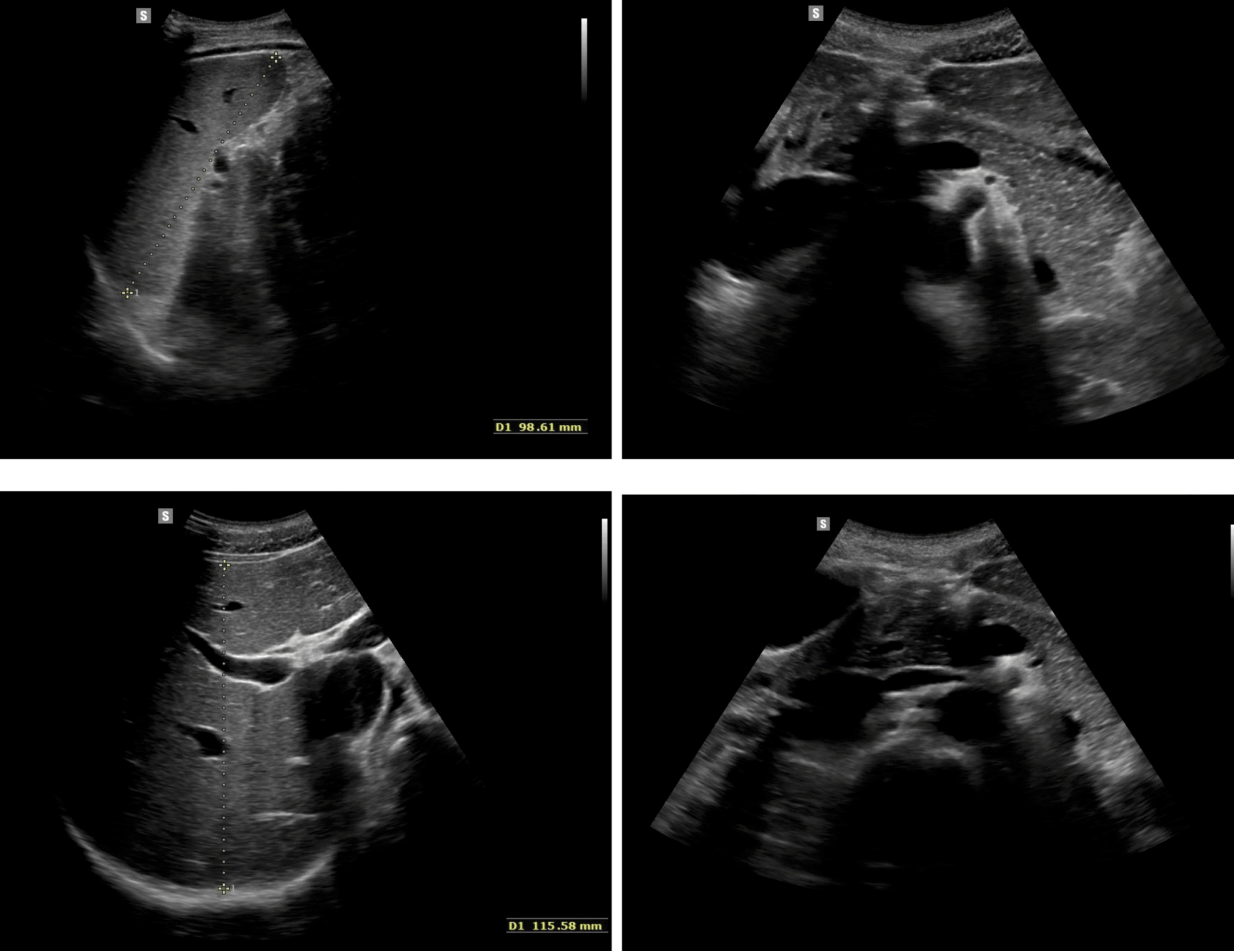
Ultrasound images of the abdomen Upper Right Image – Spleen: The spleen is of normal size (bipolar measurement: 99 mm) and exhibits a homogeneous echotexture without focal lesions. Lower Left Image – Liver: The liver is not enlarged (right lobe AP dimension: 116 mm), with a homogeneous structure and normal echogenicity. No focal lesions are present. The portal vein is of normal width. Upper and Lower Right Images – Upper Abdominal Structures: This view demonstrates normal-appearing upper abdominal structures. The pancreas appears normal in size and echotexture, with no focal lesions. The Wirsung duct is not dilated. No enlarged lymph nodes or abnormal retroperitoneal findings are observed.

**Figure 2:**
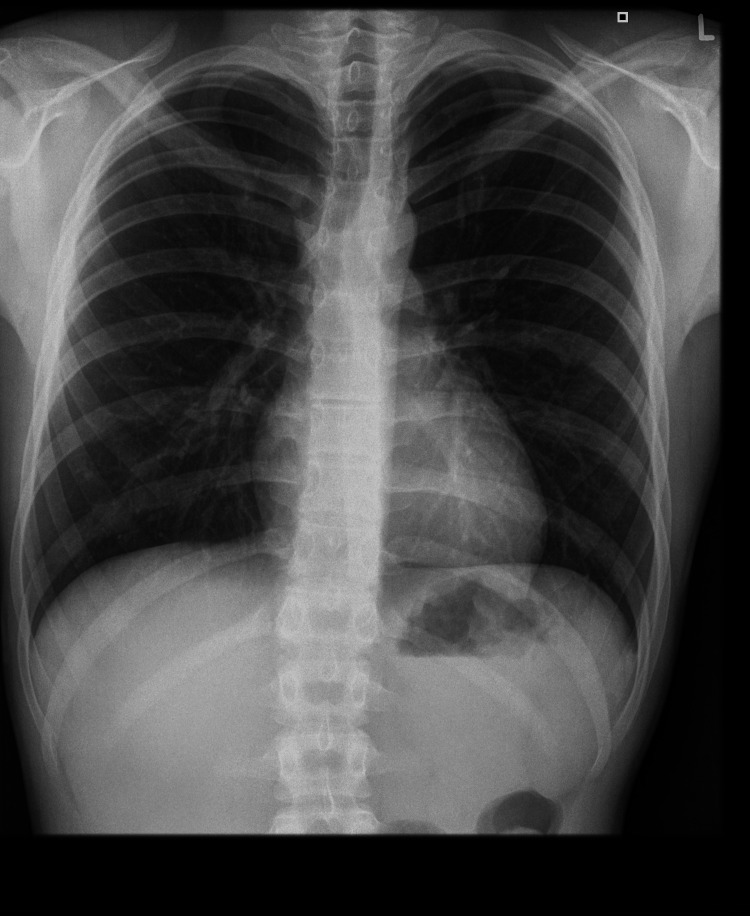
Normal chest posteroanterior (PA) X-ray

On admission, the patient's general condition was good, weight was 56.08 kg (10-25 percentile), height was 173 cm (10-25 percentile), and BMI was 16.9 (75-90 percentile). No significant deviations were observed on physical examination. On the day of admission, laboratory tests, abdominal ultrasound (Figure 1), chest X-ray (Figure 2), lactose test, and fecal calprotectin levels were normal. However, in the afternoon, repeated vomiting was observed, initially with bile, then with large amounts of saliva, accompanied by agitation, profuse sweating, motor restlessness, and hand tremors; the patient was also aggressive toward his mother and medical staff. Attention was drawn to the boy's inadequate behavior: fear, calling for his mother, then aggression, and declaring a desire to escape from the ward. For this reason, urine toxicology tests were ordered, in which the presence of tetrahydrocannabinol was confirmed. Upon conducting a more thorough investigation, the patient confirmed the use of the substance a few days before admission to the clinic. A conversation was held with the mother regarding the child's use of psychoactive substances, and she declared that she knew about the problem and for this reason, attended a psychologist with the child. Each time the onset of symptoms occurred after ingestion of food xenobiotics (including alcohol and marijuana), the patient confirmed the temporal relationship between the onset of symptoms and ingestion of xenobiotics. In addition, a history of cigarette smoking is currently denied. The patient confirmed a temporal relationship between psychoactive substance use and alcohol consumption.

**Figure 3:**
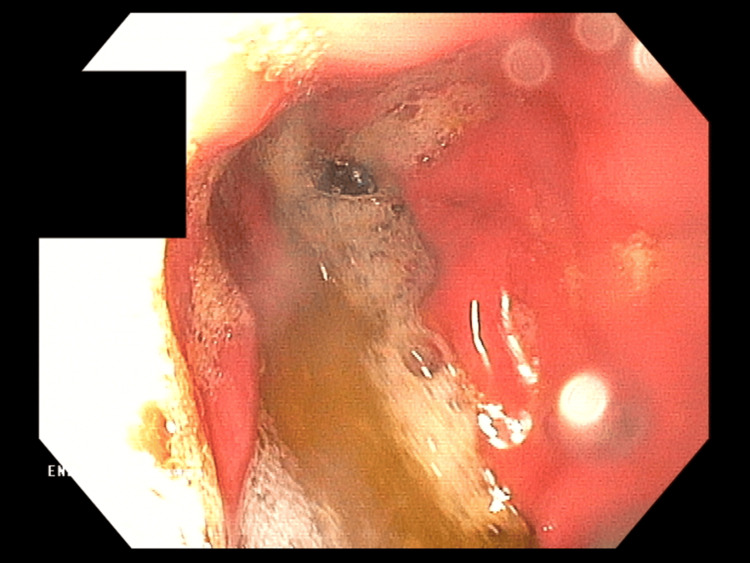
Gastrofiberoscopy demonstrating erosive gastritis

Symptomatic treatment (intravenous hydration, analgesic, and antiemetic treatment) was administered in the ward with good results. In the following days, a gradual resolution of symptoms was observed; the patient ate and drank willingly, did not report any complaints, and passed stools normally. During hospitalization, the diagnosis was expanded with gastrofiberoscopy, where erosive gastritis was described, and the Helicobacter pylori urease test result was negative (Figure 3). MRI of the head was performed, which was normal. In addition, the patient was screened for several metabolic diseases in various tests, including CoA medium-chain fatty acid dehydrogenase deficiency, tyrosinemia, maple syrup disease, hyperphenylalaninemia, and disorders of other amino acids metabolism.

In view of the whole clinical outcome, excluding all other potential causes of vomiting mentioned above, the boy was diagnosed with cyclic vomiting syndrome provoked by xenobiotic ingestion according to the Rome IV criteria. An absolute ban on alcohol and other psychoactive substances, urgent consultation with a child psychiatrist, and constant psychological care were recommended. Currently, the patient is feeling well and vomiting has not been observed for five months. According to the boy, he has not used marijuana since his hospitalization.

## Discussion

Epidemiology

In the United States, more than 3500 teenagers experiment with cannabis daily, surpassing cigarette initiation by over twofold [1]. Among U.S. youth aged 12-17, 13.2% used cannabis at least once in 2019, with 2.8% meeting the Diagnostic and Statistical Manual of Mental Disorders, Fourth Edition (DSM-IV) criteria for cannabis abuse or dependence [1]. Research from 2019 on illicit drug consumption among European youth has revealed a significant prevalence of psychoactive substance use. Studies indicate that 17% of teenagers in Europe have experimented with illegal drugs at least once [2]. The situation in Poland is even more concerning, with 22% of adolescents admitting to using prohibited substances at some point in their lives [2]. Cannabis emerged as the most commonly used illegal drug among teenagers, maintaining a steady 16% prevalence rate across Europe since 2015, while in Poland, this figure stands at 21% [2]. Worth noting is the fact that on November 1, 2017, Poland's legislation regarding herbal cannabis underwent a significant change. Cannabis was reclassified as a legal pharmaceutical raw material for the production of prescription medications. Medical professionals since then are permitted to prescribe cannabis following the same protocols as other controlled substances [3]. The 2023 statistics demonstrate the enduring appeal of marijuana and the expansion of the cannabis clinic sector. Compared to the year 2022, the quantity of issued prescriptions saw a more than twofold increase. Even more noteworthy, the overall volume of cannabis prescribed reached 4.6 tons, nearly quadrupling the amount prescribed in 2022 [4]. What’s more, the average Delta-9-tetrahydrocannabinol (THC) content in cannabis products has increased significantly, rising from approximately 3% in the 1960s to over 20% in the early 2000s [5]. This fact, coupled with increased consumption among adolescent users during the COVID-19 pandemic, suggests that today's youth are ingesting higher quantities of THC compared to previous generations [6].

Pathophysiology

The Endocannabinoid System

The endocannabinoid system (ECS) consists of the cannabinoid type 1 (CB1) and cannabinoid type 2 (CB2) receptors, which are G-protein coupled receptors that act by inhibiting adenylate cyclase. Anandamide (AEA) and 2-AG (arachidonolylglycerol) are their endogenous ligands, derived from arachidonic acid [7]. CB1 receptors are distributed across various organ systems, including the cardiovascular, immune, respiratory, and urinary systems [8]. However, their primary areas of influence are within the digestive and nervous systems [8]. The vagus nerve serves as a pathway for CB1 receptors to affect the hypothalamus-pituitary-adrenal gland (HPA) axis. These receptors are also located in the cerebellum, hippocampus, and hypothalamus. Within the hypothalamus, CB1 receptors play a role in regulating body temperature [8]. CB2 receptors have a more restricted distribution, primarily occurring in the ileum and on lymphoid cells. In these locations, they play a role in controlling immune cell movement, the release of cytokines, and the modulation of pain responses. The ECS contributes to several gastrointestinal processes, including the management of gastric acid release and intestinal motility, the suppression of inflammatory responses, and the regulation of visceral pain transmission [8]. Cannabinoids' antiemetic effects primarily target the nucleus tractus solitarius (NTS) in the medulla. Within this region, the ECS employs CB1 receptors to provide negative feedback under normal physiological conditions, thereby diminishing the impulse to vomit. NTS neurons also express transient receptor potential vanilloid-1 receptors (TRPV1), which exhibit contrasting effects to those of CB1 receptors [9]. Substance P, the pain and nausea mediator, is also controlled by TRPV1. Small amounts of cannabis exhibit an anti-nausea effect by enhancing the ECS's negative feedback on the NTS, resulting in its application for managing nausea and vomiting induced by chemotherapy [7]. Recent research has proposed various mechanisms to explain the seemingly contradictory pro-emetic effects of long-term cannabis use in CHS.

Cannabinoid Re-intoxication and Effects on Cannabinoid Receptors

Tetrahydrocannabinol, the main psychoactive ingredient in cannabis, is a fat-soluble substance that accumulates in adipose tissue during long-term use, extending its effects on CB1 and CB2 receptors. When the body experiences physiological stress or fasting, adrenocorticotropic hormone (ACTH) triggers lipolysis, which can release stored THC back into the bloodstream. This released THC can bind to ECS receptors, augmenting recent cannabis effects and potentially causing renewed intoxication in chronic users. Elevated THC levels at receptor sites can trigger or exacerbate nausea and vomiting [1,7]. A possible explanation is that prolonged exposure to endogenous cannabinoids leads to the downregulation and desensitization of CB1 receptors in the brain, which subsequently inhibits the typical antiemetic function of the endocannabinoid system (ECS). This alteration may then make the patient susceptible to hyperemesis during periods of stress when, in addition to re-intoxication, the hypothalamic-pituitary-adrenal (HPA) axis triggers increased vagal nerve activity, resulting in emesis [10]. Furthermore, gastric emptying is slowed by CB1 receptor activation in the enteric nervous system [7].

Prolonged cannabinoid use also alters the HPA axis through CB1 receptors, resulting in impaired endogenous temperature regulation and subsequent hypothermia, which can trigger nausea and vomiting [9]. This phenomenon may explain why individuals feel relief bathing in hot water (hydrothermotherapy), a practice that induces vasodilation and redirects blood flow from the digestive system to the skin [10]. Thermoregulation is also influenced by TRPV1 receptors, which stimulate the release of Substance P. During periods of chronic stress, the upregulation of TRPV1 and downregulation of CB1 receptors may result in heightened visceral pain sensitivity, leading to nausea and emesis. This condition can be further exacerbated by THC re-intoxication, which occurs because of stress-induced lipolysis [11].

Diagnosis

CHS progresses through three distinct stages: the initial prodromal phase, followed by the hyperemesis phase, and concluding with the postdrome phase. The prodromal stage, which may extend over several months, is marked by discomfort in the abdomen, loss of appetite, and feelings of nausea. Despite these symptoms, individuals can typically maintain their regular eating patterns during this period. The subsequent hyperemesis phase is characterized by recurrent vomiting, reminiscent of cyclical vomiting syndrome. This acute vomiting stage generally lasts for one to two days, while abdominal pain, concentrated in the umbilical and surrounding areas, may persist for approximately 10 days [12]. It is during this phase that patients typically seek medical attention due to the severity of their symptoms, and it is also when most CHS-related complications arise. The final phase occurs after patients discontinue cannabis use, although CHS may reoccur if cannabis consumption is resumed [13].

While the Rome IV criteria are frequently used to identify early CHS in adults, their applicability to younger populations remains uncertain. It is not clear whether these criteria are suitable for diagnosing children and adolescents. Certain aspects of these criteria, like the length of prolonged cannabis use and the timing of its discontinuation, remain controversial and require further clarification [7]. The Rome IV adult criteria are presented in Table 1. Although frequent hot bathing may be associated with CHS, it should not be considered a conclusive or reliable diagnostic sign.

**Table 1:** Rome IV criteria for the diagnosis of CHS in adult patients Adapted from [14] under Creative Commons Attribution (CC BY 4.0) CVS: cyclic vomiting syndrome; CHS: cannabinoid hyperemesis syndrome

Essential criteria				
1.	Recurrent vomiting episodes with a stereotypical pattern resembling CVS — similar in terms of onset, duration, and frequency.			
2.	Symptoms develop following a period of prolonged, heavy cannabis use.			
3.	Vomiting episodes resolve with sustained cannabis cessation.			
Note	Symptoms must have persisted for at least the past three months, with the first onset occurring a minimum of six months before diagnosis.			
Additional supportive observations				
Some patients exhibit compulsive hot bathing behavior (taking unusually long hot showers or baths to ease symptoms).				

The childhood Rome IV criteria don’t distinguish disease entities such as CHS. Only criteria for cyclic vomiting syndrome (CVS) are available for clinical use in these situations, though chronic use of cannabis is mentioned as a potential factor associated with symptom causing [15]. Some authors propose a more pragmatic set of diagnostic criteria for pediatric patients, intended as a practical guide for practitioners in the clinical environment [14,16]. These criteria are presented in Table 2.

**Table 2:** Suggested pragmatic diagnostic criteria for pediatric and adolescent cannabis hyperemesis Adapted from [14] under Creative Commons Attribution (CC BY 4.0) CVS: cyclic vomiting syndrome

Primary criteria (Essential)				
1.	Cannabis use on a regular basis for at least three months			
2.	Development or worsening of repeated nausea and vomiting episodes, with characteristics resembling CVS, after regular cannabis use begins			
3.	Exclusion of other medical causes through appropriate diagnostic evaluation, with all relevant investigations returning negative results.			
Additional supportive features				
a.	Symptom relief achieved through hot showers or baths			
b.	Unintentional weight loss			
c.	Abdominal discomfort or pain			
d.	Alterations in bowel habits			

Management

The initial approach, which should be implemented concurrently with ongoing diagnostic efforts, focuses on supportive care. This includes administering intravenous (i.v.) fluids for rehydration, addressing electrolyte imbalances, and providing a common antiemetic like ondansetron. It's important to note that approximately one-third of CHS patients will experience symptom improvement with standard antiemetic therapy, potentially serving as an early diagnostic indicator [1]. The sole known, effective, long-term solution for CHS is the complete discontinuation of cannabis use. While various management approaches are currently utilized for CHS, definitive first and second-line treatment options have yet to be established [12]. In the treatment of CHS, worth considering is haloperidol, which in a 0.05 mg/kg dose showed no extrapyramidal side effects while it had superior effects over 8 mg ondansetron in reducing both abdominal pain and nausea in CHS patients with active vomiting [17]. There is also the positive effect of benzodiazepines, the most used drugs among teenagers with active CHS, and capsaicin in an ointment that is available in Poland without a prescription. Research has shown that various medications, including promethazine, metoclopramide, dimenhydrinate, opioids, proton pump inhibitors, and histamine2-receptor antagonists, are not effective in treating CHS [18].

Considering the lack of effective drug-based treatments for cannabis cessation, urgent constant psychological and psychiatric care becomes crucial in tackling this aspect of patient care.

## Conclusions

Cannabis hyperemesis syndrome (CHS) presents a complex diagnostic and treatment challenge, particularly in pediatric and adolescent populations. The increasing prevalence of cannabis use among youth and rising THC potency underscore the importance of recognizing and managing this condition. Our patient suffered from recurrent vomiting for a relatively long time, but the impact of xenobiotic ingestion was not considered during the diagnosis, as ingestion is not a common cause of vomiting in children. However, due to the increasing trend of marijuana consumption among teenagers, it is crucial to consider CHS in the differential diagnosis for recurrent vomiting in adolescents with chronic cannabis use, relying on a thorough history and symptom recognition for diagnosis and focusing on supportive care and cannabis cessation for management. A multidisciplinary approach and increased awareness among healthcare providers are crucial for the effective treatment and prevention of CHS. The growing importance of the diagnostic problem in the pediatric population suggests the need to list CHS as a separate entity with its own diagnostic criteria in the next edition of the Rome criteria.

## References

[1] Lonsdale H, Wilsey MJ (2022). Paediatric cannabinoid hyperemesis. Curr Opin Pediatr.

[2] Engelgardt P, Krzyżanowski M, Borkowska-Sztachańska M, Wasilewska A, Ciucias M (2023). Life time use of illicit substances among adolescents and young people hospitalized in psychiatric hospital. Sci Rep.

[3] Hordowicz M, Jarosz J, Czaplińska M, Leonhard A, Klimkiewicz A (2021). Polish physicians’ perspectives on medical cannabis policy and educational needs: results of an online survey. J Clin Med.

[4] (2025). 3 months after the regulation - what has changed on the “marihuanomat” market? [In Polish]. https://remedium.md/publikacje/opinie/3-miesiace-po-rozporzadzeniu-co-zmienilo-sie-na-rynku-marihuanomatow.

[5] Murray RM, Quigley H, Quattrone D, Englund A, Di Forti M (2016). Traditional marijuana, high-potency cannabis and synthetic cannabinoids: increasing risk for psychosis. World Psychiatry.

[6] Lonsdale H, Brown JM, Wilsey M (2022). Adolescent cannabis hyperemesis syndrome during the COVID-19 pandemic. Pediatr Emerg Care.

[7] Perisetti A, Gajendran M, Dasari CS (2020). Cannabis hyperemesis syndrome: an update on the pathophysiology and management. Ann Gastroenterol.

[8] Galli JA, Sawaya RA, Friedenberg FK (2011). Cannabinoid hyperemesis syndrome. Curr Drug Abuse Rev.

[9] DeVuono MV, Parker LA (2020). Cannabinoid hyperemesis syndrome: a review of potential mechanisms. Cannabis Cannabinoid Res.

[10] Burillo-Putze G, Richards JR, Rodríguez-Jiménez C, Sanchez-Agüera A (2022). Pharmacological management of cannabinoid hyperemesis syndrome: an update of the clinical literature. Expert Opin Pharmacother.

[11] Gunasekaran N, Long LE, Dawson BL (2009). Reintoxication: the release of fat-stored delta(9)-tetrahydrocannabinol (THC) into blood is enhanced by food deprivation or ACTH exposure. Br J Pharmacol.

[12] Khalid N, Abdullah M, Khalil M, Afzal MA, Hindawi M (2023). Cannabis hyperemesis syndrome in a young patient: a case report and literature review. Cureus.

[13] Stumpf JL, Williams LD (2021). Management of cannabinoid hyperemesis syndrome: focus on capsaicin. J Pharm Pract.

[14] Drossman DA, Tack J (2022). Rome Foundation clinical diagnostic criteria for disorders of gut-brain interaction. Gastroenterology.

[15] Hyams JS, Di Lorenzo C, Saps M, Shulman RJ, Staiano A, van Tilburg M (2016). Childhood functional gastrointestinal disorders: child/adolescent. Gastroenterology.

[16] Lonsdale H, Kimsey KM, Brown JM, Dey A, Peck J, Son S, Wilsey M (2021). Pediatric cannabinoid hyperemesis: a single institution 10-year case series. J Adolesc Health.

[17] Furyk JS, Meek RA, Egerton-Warburton D (2015). Drugs for the treatment of nausea and vomiting in adults in the emergency department setting. Cochrane Database Syst Rev.

[18] Zhu JW, Gonsalves CL, Issenman RM, Kam AJ (2021). Diagnosis and acute management of adolescent cannabinoid hyperemesis syndrome: a systematic review. J Adolesc Health.

